# Size and shape in the evolution of ant worker morphology

**DOI:** 10.7717/peerj.205

**Published:** 2013-11-05

**Authors:** Marcio R. Pie, Marcel K. Tschá

**Affiliations:** Laboratório de Dinâmica Evolutiva e Sistemas Complexos, Departamento de Zoologia, Universidade Federal do Paraná, Curitiba, Brazil

**Keywords:** Lines of least resistance, Allometry, Caste, Morphological innovation

## Abstract

Morphological evolution in ants has been traditionally thought as being strongly influenced by selection for colony ergonomic efficiency. Although many studies have focused on the evolution of social characteristics in ants, little is known about the evolution of worker morphology at a macroevolutionary scale. In this study, we investigate the tempo and mode of the evolution of worker morphology, focusing on changes in size and shape. Our datasets included a large sample of species from different ant genera, as well as variation within the hyperdiverse genus *Pheidole*, for a total of 1650 measurements. The rate of size evolution was at least five times faster than the rate of shape evolution. The fit of alternative models of morphological evolution indicated statistically significant phylogenetic signal in both size and shape and in all datasets. Finally, tests of rate heterogeneity in phenotypic evolution among lineages identified several shifts in rates of evolution in both datasets, although the timing of shifts in size and shape was usually not concordant.

## Introduction

The organization of the worker caste in social insects that has intrigued naturalists for centuries (Darwin, 1859) is based to a large extent on division of labor, in which a variety of tasks are carried out simultaneously by different workers, leading to a high degree of ergonomic efficiency at the colony level (Oster & Wilson, 1978; Hölldobler & Wilson, 1990; Hölldobler & Wilson, 2009). The efficient orchestration of a multitude of tasks, including foraging, nest defense, and brood care, was made possible by extensive modifications to the basic body plan of solitary ancestors to allow for the production of a (mostly) sterile, multi-purpose worker force that was unconstrained by the needs associated with reproductive activities. Such modifications may include specialized head and mandible characteristics, reduced ovarian development, winglessness, and even polymorphism within the worker caste (Hölldobler & Wilson, 1990).

The evolution of worker morphology in social insects has been traditionally investigated using bivariate plots in which pairs of morphological characters are compared, seeking discontinuities in allometric relationships (Oster & Wilson, 1978; Hölldobler & Wilson, 1990; Wilson, 1953; Feener, 2009), as had been the rule in evolutionary biology since the modern synthesis (Huxley, 1932; Simpson, 1944; Rensch, 1960; Gould, 1966; Gould, 1974; Gould, 1982). The power of this method lies in its simplicity, describing in general terms the different types of caste polymorphism in ants (e.g., mono-, bi- and triphasic allometries), although in practice the distinction between different types of allometry is often arbitrary (e.g., Diniz Filho et al., 1994). However, recent advances in the statistical modeling of morphological traits (e.g., Magwene, 2001) have provided unprecedented opportunities to understand patterns of modularity and integration in the organization of trait variation. The first study to apply these methods to investigate morphological evolution in the worker caste was conducted by Pie & Traniello (2006) using as a model system the ant genus *Pheidole* – a highly diverse and abundant group of ants that are distributed worldwide in tropical and subtropical regions. The worker caste of *Pheidole* ants is typically divided into major workers specialized in food processing or colony defense and minor workers responsible for quotidian colony tasks. By investigating morphological variation in minor and major workers of a comprehensive sample of *Pheidole* species, Pie & Traniello (2006) showed that major worker morphology is not a simple extrapolation of minor morphology and that patterns of trait covariation are apparent even between traits that are distant from one another in the worker’s body. These high levels of morphological integration might hamper changes in shape because of possibly undesirable correlated responses in trait evolution. Such constraints might not be so severe in the case of variation in worker size, given that larger or smaller bodies could be generated by simply changing the stopping points along growth trajectories without necessarily affecting trait covariation. However, little is known about the relative importance of size and shape changes during the evolution of the worker caste.

The goal of the present study is to use phylogenetic comparative methods to assess the relative importance of size and shape changes during the evolution of the worker caste. Two large datasets were compiled to describe morphological variation across a large sample of ant lineages, as well as within *Pheidole* major and minor castes. Substantial differences were uncovered in the evolution of size and shape among datasets, suggesting that the evolution of caste dimorphism might have been associated with a drastic shift in the way size and shape affect worker evolution in ants.

## Materials and Methods

Two comprehensive datasets were used in the present study, representing two levels of divergence among ant lineages. The first included 115 ant species, which were selected to encompass a taxonomically diverse sample based on the phylogeny by Moreau et al. (2006), including representatives of 115 genera from 18 subfamilies. Morphological measurements were obtained from images of the species in Moreau et al. (2006) available in Antweb (http://www.antweb.org/), using tpsDig 2.05 (Rohlf, 2005). Whenever a species was missing from Antweb, or when the species in the phylogeny was only identified to the genus level, a congener was selected to be measured and then replaced the original species in the dataset. Although this replacement was necessary in 60% of the species, these differences should not affect our results, as long as the involved genera are monophyletic. Morphological measurements included: minimum distance between the eyes in full-face view (BE), minimal distance between antennal sockets (BA), maximum head width (HW), maximum head length in full-face view (HL), width of the mesossoma (MW), and length of the mesossoma from the occiput to the end of the propodeum (ML). If a species showed caste polymorphism, the minor caste was measured. The second dataset morphometric included information obtained directly from the measurements on the type specimens of 96 New World *Pheidole* species available in Wilson (2003) that were also present in the recent phylogeny by Moreau (2008) and included five traits (measured separately for minors and majors): HL, HW, MW, scape length (SL), and eye length (EL). Given that the phylogeny provided by Moreau (2008) is not ultrametric, we removed its outgroups and carried out penalized likelihood analysis (lambda = 1; Sanderson, 2002) to obtain relative divergence times for later analyses. Although measurements from single specimens were used in all cases, we assume that intraspecific variation in morphological traits is small in relation to interspecific variation as commonly used in comparative analyses (see Pie & Traniello, 2006). All datasets were ln-transformed prior to the analyses to improve the normalization of variances for a total of 1650 measurements.

A principal component analysis (PCA) was carried out on the covariance matrix of each dataset, with the resulting biplot being described using the phylomorphospace function in the R package phytools (Revell, 2012). The scores of the species on each PC were used to test for the level of phylogenetic signal using the model developed by Pagel (1999) based on an extension of a constant-variance random-walk model (sometimes called Brownian motion). Under those conditions, the degree of similarity in a given trait between two lineages is proportional to the extent of their shared history, as indicated by the phylogeny, such that traits evolve at each instant of “time” at with a mean character change of zero and an unknown but constant variance σ^2^. Pagel introduced another parameter, λ, to estimate the extent to which the phylogeny correctly predicts patterns of similarity among species. This parameter can range from 1 (as predicted by the Brownian motion model) to 0 (trait similarity among species is independent of phylogeny). Also, the relative fit of a constant-rate (Brownian) model was tested against an alternative model in which trait evolution can be concentrated near the base of the phylogeny or near the tips, as measured by a scaling parameter δ being estimated to be <1 or >1, respectively. (Exploratory tests using the analogous “early burst” model (Blomberg, Garland & Ives, 2003) showed the same qualitative results and are here omitted for the sake of brevity.) Hypothesis testing was based on the likelihood-ratio statistic, which compares the goodness of fit of a model to the data with that of a simpler model that lacks one or more of the parameters. Analyses using Pagel’s method were implemented using geiger (Harmon et al., 2008). Finally, we tested for shifts in the rates of evolution among lineages using the transformPhylo.ML function in motmot (Thomas & Freckleton, 2012). The tm1 algorithm used in this approach proceeds according to the following workflow: (1) evaluate the likelihood of a single-rate Brownian model; (2) fit rate heterogeneous models and evaluate the likelihood at each node in the phylogeny (one at a time) where the second rate is permitted in all branches descended from the focal node plus the stem branch leading to that node (new shifts are inferred only as monophyletic groups); (3) select the best-fitting two-rate model; (4) fit rate heterogeneous models with two-rate shifts where one of the shifts must occur at the node identified in step 3 (each additional shift defines a monophyletic set of taxa but rate shifts can be nested); (5) continue this procedure until a user-defined maximum number of rate shifts has been reached (8 in our case); (6) select the preferred model from the set of best-rate heterogeneous models using the Akaike Information Criterion (AIC). There is a second algorithm in motmot (tm2) that also allows for shifts in single branches of the tree (as opposed to the exclusively clade-wide shifts of tm1), but the results for our datasets were nearly identical to those of tm1 and were omitted from later consideration.

## Results

**Figure 1:**
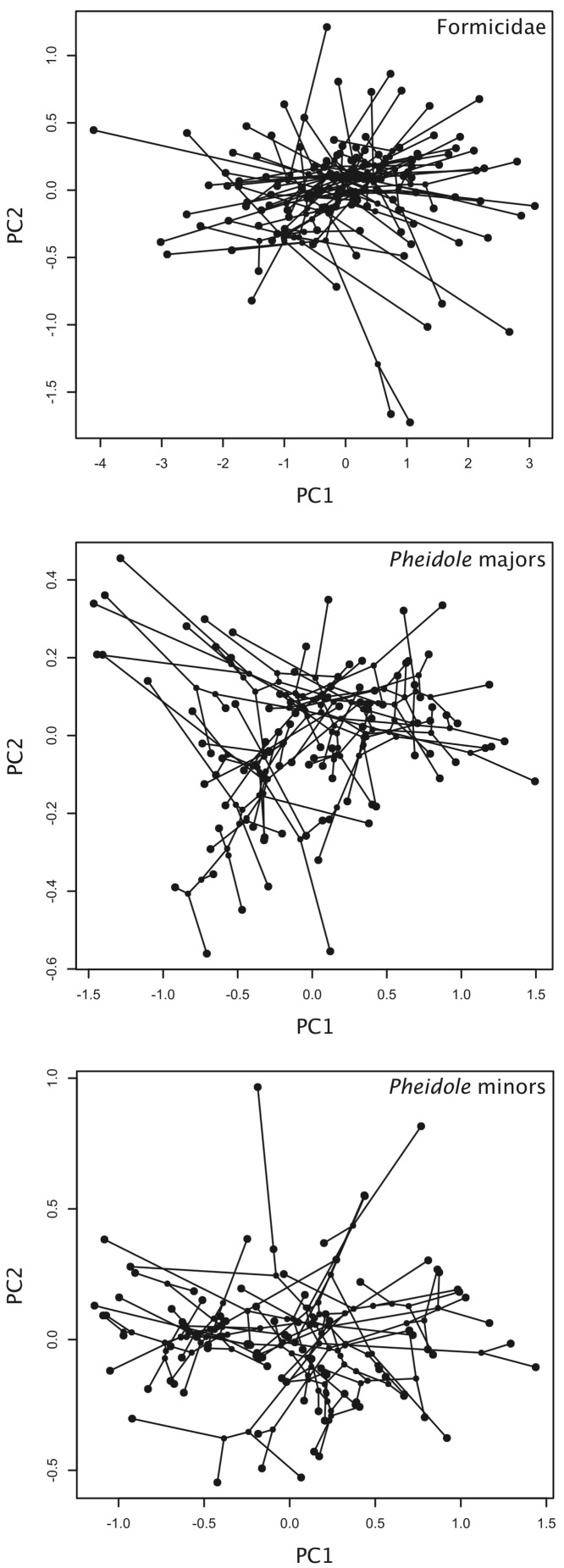
Phylomorphospaces based on PCA scores. Phylomorphospaces described by the first and second principal components of morphological variation in ants.

In all PCAs, the loadings on the first PC were of similar magnitude and the same direction and were therefore interpreted as a size axis, whereas the remaining axes were interpreted as reflecting different aspects of worker shape (Table 1, Fig. 1). More than 80% of the variance was concentrated on the first PC, indicating that the rate of size variation in ants is more than five times larger than the rate of shape evolution. The second PC was investigated further as a proxy for the main direction of shape variation in each dataset, given that it encompassed nearly half of the remaining variance (i.e., between 8 and 14%, Table 1). In the dataset that included a variety of ant lineages, the second PC reflected mainly the negative relationship between minimal distance between antennal sockets and length of mesosoma + head length. In the *Pheidole* datasets, the loadings on second PC differed between major and minor workers. In minors, it indicated the negative association between scape length + eye length and mesosoma width + head width + head length, whereas in majors it reflected most strongly the negative association between eye length and scape length.

**Table 1:** Principal Component Analyses of ant morphological traits.

	Variable	PC1	PC2	PC3	PC4	PC5
*Pheidole* Minors	HW	−0.29	0.23	−0.58	0.25	0.69
	HL	−0.38	0.23	−0.13	0.67	−0.58
	SL	−0.60	0.37	0.66	−0.14	0.23
	EL	−0.55	−0.84	−0.04	−0.02	0.02
	MW	−0.34	0.24	−0.47	−0.69	−0.37
	**% variance**	**0.80**	**0.14**	**0.05**	**0.01**	**<0.01**
*Pheidole* Majors	HW	−0.45	0.37	−0.21	−0.77	−0.13
	HL	−0.43	0.41	0.07	0.31	0.74
	SL	−0.49	−0.57	−0.63	0.18	0.01
	EL	−0.46	−0.47	0.74	−0.15	−0.01
	MW	−0.40	0.39	0.10	0.50	−0.66
	**% variance**	**0.84**	**0.08**	**0.05**	**0.02**	**0.01**
Formicidae	HW	0.39	−0.13	0.18	0.10	−0.47
	HL	0.37	−0.29	−0.28	−0.79	−0.18
	BE	0.40	−0.11	0.78	−0.11	0.44
	BA	0.47	0.87	−0.16	−0.06	0.06
	MW	0.39	−0.15	0.03	0.49	−0.48
	ML	0.42	−0.33	−0.51	0.33	0.57
	**% variance**	**0.88**	**0.08**	**0.02**	**0.01**	**0.01**

There was strong support for the existence of phylogenetic signal for both size and shape in all datasets (Table 2). In addition, the rate of size evolution appears to proceed at a constant rate over time, both for ants as a whole and for *Pheidole*, given that adding the δ parameter did not improve the fit of the model to the data in relation to a simpler constant-rate model. However, intriguing differences among datasets were detected with respect to shape. In ants in general, the maximum likelihood estimate δ was 0.325, indicating that shape evolution was accelerated in the early stages of ant evolution. On the other hand, the maximum likelihood estimates of δ for the entire *Pheidole* dataset were 5.75 and 3.54 for minors and majors, respectively, suggesting the opposite trend in relation to ants as a whole – an accelerating rate of shape evolution. However, it is important to note that, even though the *Pheidole* dataset was based on measurements of type specimens by Wilson (2003), some of the species such as *P. crassicornis, P. davisi, P. umphreyi, P. boltoni* and *P. adrianoi* appear to be outliers and might actually contain measurement errors. In fact, omitting these species decreases substantially the estimates of δ, although this effect is possibly confounded by a reduced power due to a smaller sample size. Nevertheless, our estimates of δ > 1 in *Pheidole* should be interpreted with caution until more measurements from these species are obtained.

**Table 2:** Fit of alternative models of trait evolution, based on the method of Pagel (1999).

	*Pheidole*				Formicidae	
	MAJORS		MINORS			
	PC1	PC2	PC1	PC2	PC1	PC2
lik λ	−64.53	32.14	−50.72	9.28	−203.247	−48.273
$\hat {\lambda }$	0.99	0.79	0.98	0.78	1.05	1.03
lik λ = 0	−96.009	17.176	−88.359	−4.220	−203.247	−67.567
*p*	2.11E−15	4.49E−08	4.07E−18	2.03E−07	0.0003	5.23E−10
lik BM	−64.564	18.815	−51.138	−10.092	−197.800	−48.825
lik δ	−62.907	24.716	−50.784	−3.686	−197.651	−46.3647
$\hat {\delta }$	0.32	3.54	0.60	5.75	1.276	0.325
*p*	0.069	6e−04	0.400	3e−04	0.584	0.026

**Figure 2:**
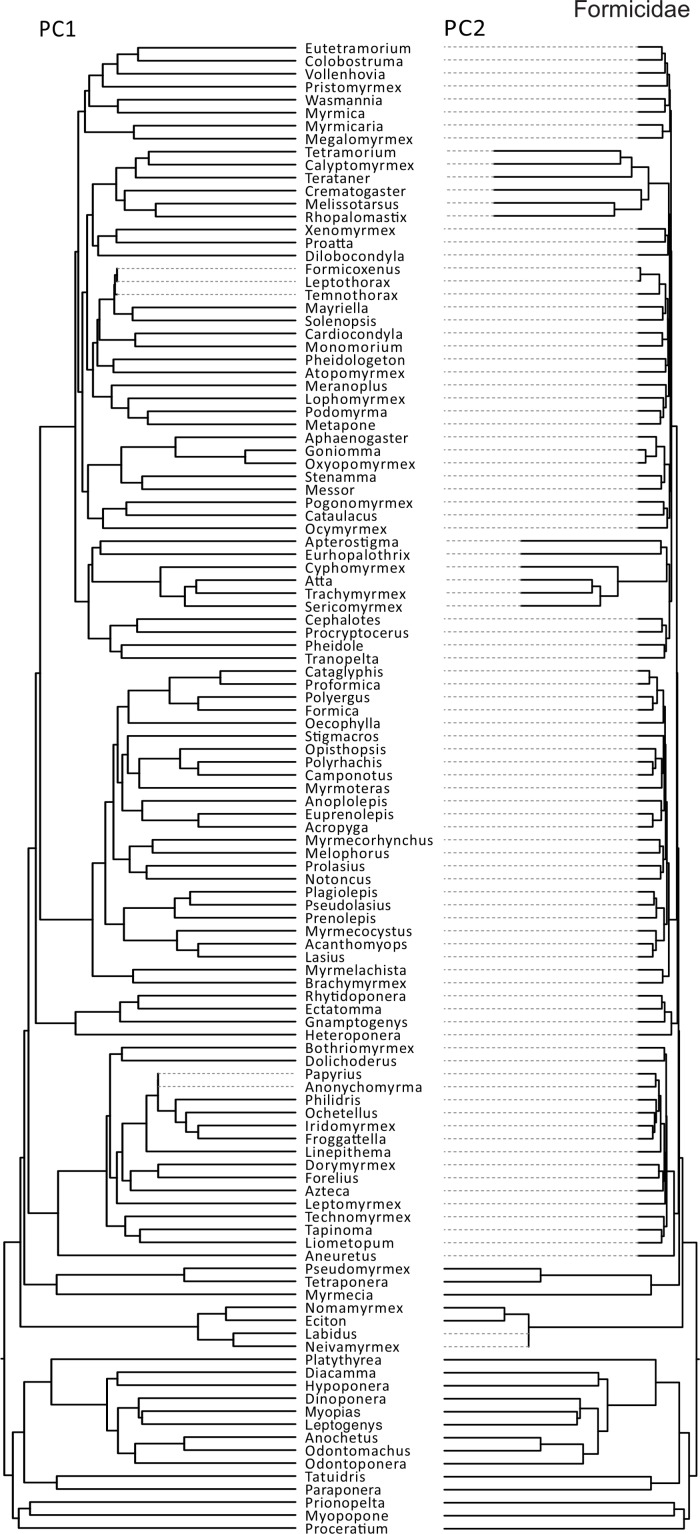
Rates of ant size and shape evolution. Phylogeny of Formicidae used in the present study in which branch lengths are transformed according to the relative rate of evolution of PCA scores. Total tree depth was rescaled to 1 to facilitate the visualization of relative rates within a phylogeny.

**Figure 3:**
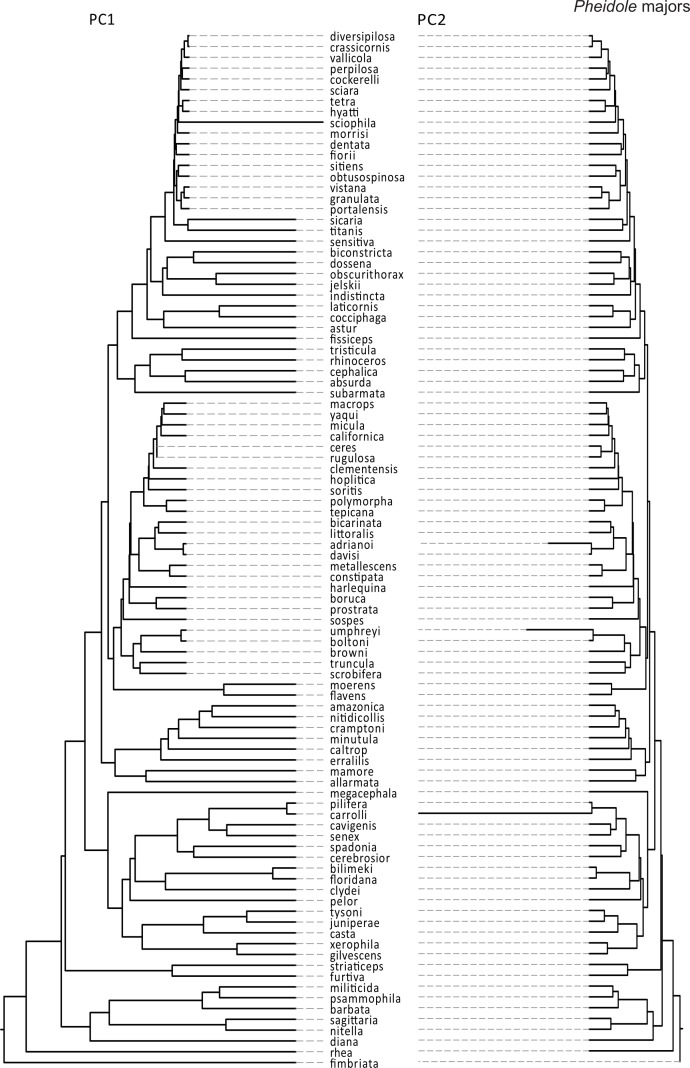
Rates of size and shape evolution in Pheidole majors. Phylogeny of Pheidole used in the present study in which branch lengths are transformed according to the relative rate of evolution of PCA scores of major workers. Total tree depth was rescaled to 1 to facilitate the visualization of relative rates within a phylogeny.

**Figure 4:**
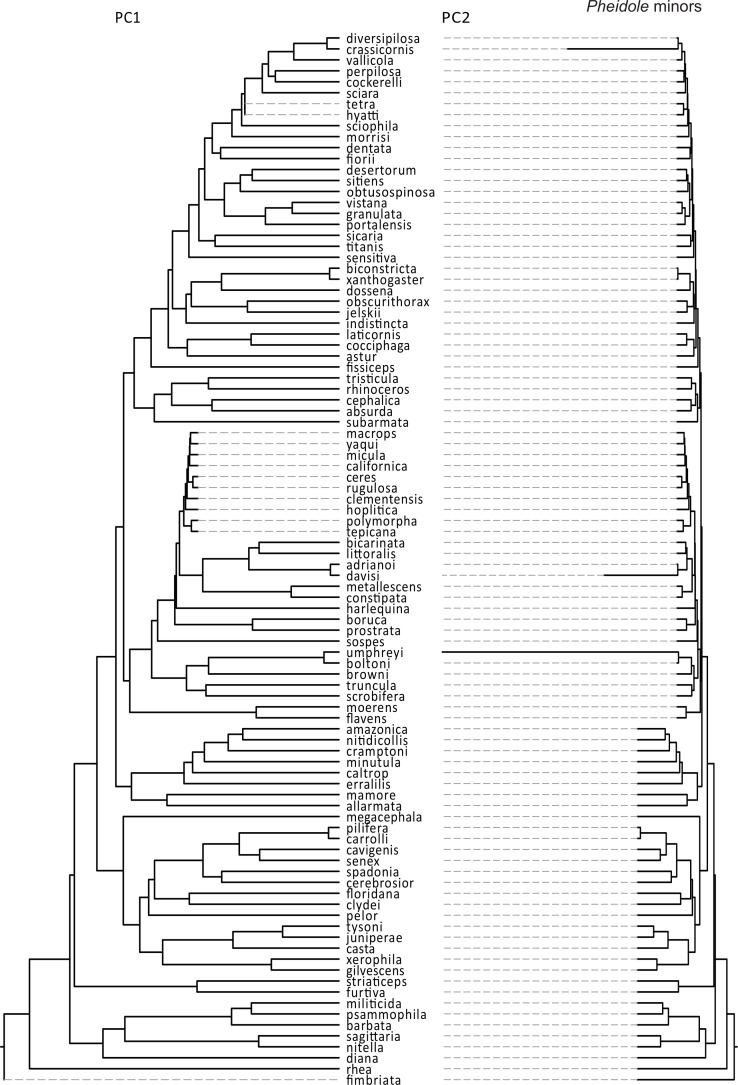
Rates of size and shape evolution in *Pheidole* minors. Phylogeny of *Pheidole* used in the present study in which branch lengths are transformed according to the relative rate of evolution of PCA scores of minor workers. Total tree depth was rescaled to 1 to facilitate the visualization of relative rates within a phylogeny.

There were markedly different modes of size and shape evolution for ants in general according to the motmot analyses (Table 3). In general, most ant lineages tend to share a similar rate of size evolution, with only two shifts being detected – both involving rate decreases. The first shift involved a clade including *Formicoxenus*, *Leptothorax*, and *Themnothorax*, whereas the second shift included *Papyrius* and *Anonychomyrma*. On the other hand, there were four identified shifts in the rate of shape evolution: (1) a negative shift involving the army ant genera *Neivamyrmex* and *Labidus*; (2) a negative shift that involved a large portion of the formicoid clade; (3) a positive shift near the origin of fungus-growing ants; and (4) a positive shift involving the genera *Tetramodium, Calyptomyrmex, Terataner, Crematogaster, Melissotarsus* and *Rhopalomastix* (Fig. 2). Similar discrepancies between size and shape evolution were also detected in *Pheidole*, both for the major and minor castes (Table 3). There were two broad negative shifts in body size evolution in majors and minors, yet they are only congruent with respect to a group of desert species (e.g., *P. macrops, P. yaqui, P. ceres*). On the other hand, shape evolution tended to follow a constant rate throughout most *Pheidole* lineages, except for a few isolated species with unusually fast rates and that might involve measurement errors (see above). If those isolated lineages are omitted from consideration, *Pheidole* majors are characterized by a homogeneous rate of shape evolution, as opposed to two general rates in the case of minors (Figs. 3 and 4).

**Table 3:** Fit of alternative models of rate heterogeneity in phenotypic evolution among lineages using the tm1 algorithm implemented in motmot (Thomas & Freckleton, 2012). lik: log likelihood of each model; *k*: number of parameters in the model; AICc: corrected Akaike Information Criterion; MLRate: estimated rate of trait evolution. See text for details.

Trait	lik	*k*	AICc	MLRate
Formicidae PC1	−192.069	4	392.502	0.003
				0.017
Formicidae PC2	−27.039	6	66.857	0.175
				0.997
				0.829
				0.001
*Pheidole* minors PC1	−28.872	6	70.687	1.00E−08
				116.23
				1000
				0.558
*Pheidole* minors PC2	23.639	6	−34.333	54.484
				32.000
				29.834
				0.389
*Pheidole* majors PC1	−51.260	6	115.464	0.001
				0.127
				0.399
				1.702
*Pheidole* majors PC2	41.315	6	−69.686	61.062
				18.621
				1.00E−08
				16.391

## Discussion

A close correspondence between worker morphology, task performance, and colony-level ergonomic efficiency has been a central tenet of ant sociobiology for over 30 years (Oster & Wilson, 1978; Hölldobler & Wilson, 1990). Evidence for a link between these three factors comes mainly from studies investigating worker morphology and specialization on particular tasks (e.g., food processing (Ferster, Pie & Traniello, 2006) and colony defense (Powell, 2008)), as well as the association between variation within the worker caste and colony fitness (e.g., Beshers & Traniello, 1994; Billick, 2002). However, given the apparently obvious advantages of having specific worker castes for particular tasks, it is intriguing that specialized worker morphologies are only present in a minority of the ant genera, and the most elaborate systems, possessed by *Eciton, Atta, Daceton*, and *Pheidologeton*, still contain no more than three truly distinct forms (Oster & Wilson, 1978; Hölldobler & Wilson, 1990). One possible explanation for this conundrum suggests that behavioral specialization within the worker caste would mitigate the selective forces favoring caste proliferation (Oster & Wilson, 1978). However, this hypothesis does not address the fact that, even in the cases where morphological variation does exist, it is still relatively limited, involving to a large extent shape differences along allometric relationships (Wilson, 1953). According to the result of the present study, ant body size can evolve over five times more than body shape. Similar differences in rates of size and shape evolution have been uncovered in other organisms (Stanley & Yang, 1987; Clyde & Gingerich, 1994; Hunt, 2007; Wood et al., 2007; Dzeverin, 2008), and are often interpreted as an indication that size may be more evolutionary labile (Stanley, 1979). Our results suggest that the difference in the way size and shape evolve is not lability *per se*, given that the estimated phylogenetic signal (λ) in size was equal or greater than that of shape (Table 2). Alternatively, size has been proposed as a possible evolutionary line of least resistance (Schluter, 1996), such that evolutionary change would be more easily attained along a size axis (Marroig & Cheverud, 2005; Marroig & Cheverud, 2010). We suggest that this mechanism might also operate within colonies, such that morphological variation might be more easily attained by varying body size (and the possible allometric consequences of this change) instead of changing shape. More generally, evolution along lines of least resistance can provide an unifying principle linking caste proliferation within colonies and the evolution of worker morphologies among different lineages.

The explicit tests of heterogeneity in rates of size and shape evolution provided in the present study provide an interesting window into the tempo and mode of evolution among ant lineages. In particular, most large-scale shifts involving several lineages involved decreases in rates of evolution (Figs. 2–4). These negative shifts are not necessarily evolutionary dead-ends, given the positive shift in rate evolution associated with the origin of fungus-growing ants. A comparison of a variety of cases of adaptive radiation has suggested that a decelerating rate of evolution is relatively uncommon (see Harmon et al., 2010). In fact, the pattern observed in ants could be interpreted as evidence for an extension of the original behavioral flexibility hypothesis of Oster & Wilson (1978), such that adjustments at the colony level could affect not only the composition of the worker caste within a colony, but also the overall rate of morphological evolution for the species as a whole. Comparisons with closely-related solitary hymenopterans using explicit models of trait evolution such as those used in the present study can provide valuable tests of this conjecture.

## Supplemental Information

10.7717/peerj.205/supp-1 Supplemental Information 1 Morphological datasets investigated in the present study. Three files are included: a family-level dataset (formicidae.csv) and two datasets with measurements major and minor workers of *Pheidole*. Acronyms are described in the text.
